# Prophylaxis during multibracket appliance treatment – a survey among general dentists in Germany

**DOI:** 10.1186/s12903-025-05843-4

**Published:** 2025-04-02

**Authors:** Waldemar Petker-Jung, Sabine Ruf, Niko C. Bock

**Affiliations:** Department of Orthodontics, Justus-Liebig-University Giessen, Schlangenzahl 14, Giessen, 35392 Germany

**Keywords:** Dental prophylaxis, Multibracket appliance, Orthodontic treatment, Oral hygiene, General dentist, Questionnaire, Survey

## Abstract

**Background:**

Accomplishing good oral hygiene represents a major challenge during multibracket appliance (MBA) treatment. In Germany, children, and adolescents between 6 and 18 years of age can attend an individual prophylaxis (IP) program free of charge twice a year. As part of this, they undergo training and receive recommendations to enhance oral hygiene. However, it remains unclear whether the general dentist feels responsible for prophylaxis during MBA treatment and how they adapt the IP sessions compared to patients without fixed appliances.

**Aim:**

To assess how general dentists manage IP in children and adolescents undergoing MBA treatment.

**Method:**

A questionnaire was sent to 2744 general dental practices in the region of Hesse, Germany. Dentists were asked regarding their opinion on the responsibility for prophylaxis during MBA treatment, the oral hygiene recommendations given to those patients and how they adapt the prophylaxis sessions to special MBA needs.

**Results:**

The response rate was 37.0% (*n* = 1014). While only 8% of the respondents consider the orthodontist as primarily responsible for IP in MBA patients, the majority considers the general dentist (46%) or both, the general dentist and the orthodontist (46%) to be responsible for IP in MBA patients. The vast majority of respondents answered that compared to patients without fixed appliances, MBA patients receive different oral hygiene recommendations during IP sessions. These recommendations are mostly related to 1. a longer toothbrushing duration (> 3 min) and 2. the use of additional tools for mechanical plaque removal (interdental brushes).

**Conclusion:**

The majority of respondents perceive the general dentist as having primary or shared responsibility for IP during MBA treatment and adapt oral hygiene recommendations to the special requirements. However, the large proportion of perceived shared responsibility also suggests considerable room for improvement. Furthermore, analysis showed a large range regarding oral hygiene recommendations, indicating the need for standardized clinical guidelines.

**Supplementary Information:**

The online version contains supplementary material available at 10.1186/s12903-025-05843-4.

## Background

Components of fixed orthodontic multibracket appliances (MBA), such as brackets, bands and archwires, impede oral hygiene and promote plaque accumulation. Not only does the amount of dental plaque increase [1], but there is also a pathogenic shift in the oral microbiome [2, 3]. This increases the risk for gingivitis [4, 5] and enamel demineralizations [5, 6] in form of white spot lesions (WSL). WSL can negatively affect smile aesthetics and—in case of aggravation—tooth preservation long-term. Multiple efforts have been made to reduce these plaque-related side effects by using remineralization [7–11] and antimicrobial agents [12–14], but with limited success. Thorough removal of dental plaque through effective tooth brushing and the use of interdental cleaning aids remains essential for effective prevention [5]. However, accomplishing good oral hygiene seems to be a major challenge for young patients undergoing MBA treatment.

In Germany, children and adolescents between 6 and 18 years of age can attend an individual dental prophylaxis (IP) program free of charge twice a year at a general dental practice. The aim is to help children establish proper oral hygiene skills, and thereby preserve dental and oral health. This program has been anchored by social law, and the costs are fully covered by the social health insurance. Those IP sessions begin with an assessment of the oral hygiene status. If necessary, this is followed by education on the causes of caries and periodontal diseases and their prevention. Additionally, the patients receive practical training in oral hygiene techniques [15].

Since the introduction of the IP program in the late 1980s, children’s oral health in Germany has improved considerably with a significant decrease of caries prevalence and incidence [16, 17]. However, when it comes to patients undergoing MBA treatment, the literature still shows high WSL prevalence values of up to 68% [18–22]. Unfortunately, prophylaxis concepts specially tailored for patients undergoing MBA treatment are missing both nationally and internationally. There is a lack of literature and high-quality studies on optimal oral health care during orthodontic treatment [23], resulting in a lack of guidelines for this group of patients, which in turn could be a contributing factor for the frequent occurrence of WSLs mentioned above. Another possible issue could be that patients do not participate in IP sessions during their orthodontic treatment due to a lack of communication between orthodontists and general dental practitioners (GDPs). It is unknown to what extent GDPs continue to feel responsible for prophylaxis during MBA treatment. If the responsibility for IP during orthodontic treatment is unclear, there is a risk for the patient to be neglected.

It should be noted that plaque removal by the dental professional is not part of the IP. However, professional prophylactic procedures are effective supportive measures for plaque reduction in MBA patients [24]. Removing archwires, rubber chains, ligatures, hooks, etc. can simplify the professional tooth cleaning (PTC), but this is difficult to implement in general dental practices.

Therefore, the aims of this questionnaire study were to assess.

- GDPs’ general attitude towards IP and PTC in children and adolescents undergoing MBA treatment and
- GDPs’ possible adaption of IP and PTC sessions for MBA patients compared to patients without MBA.

## Method

A questionnaire was developed specifically for the purposes of this study and tested on seven GDPs to assess its comprehensibility and the time required for completion. Based on the feedback, the wording and the structure were edited. The completion time was less than three minutes. The latter data were excluded from the final analysis. The questionnaire did not require ethical approval since data collection was anonymous and participation was voluntary.

### Structure of the questionnaire

**Fig. 1:**
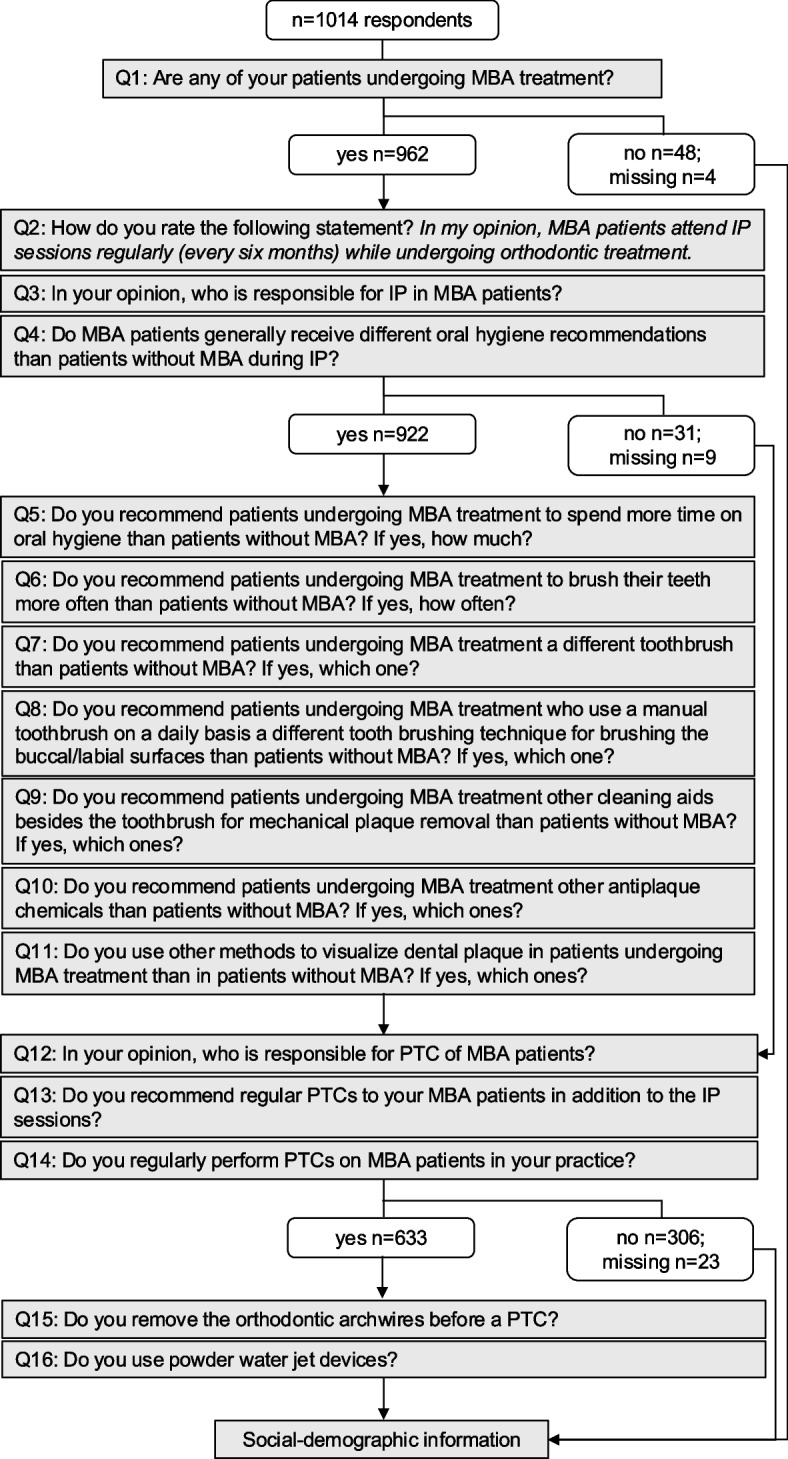
Flow diagram for branching logic of the questionnaire (see Additional file 1 for the English version and Additional file 2 for the original German wording)

The questionnaire consisted of multiple-choice questions, with the option of giving free text answers to some of the questions (see Additional file 1 for the English version and Additional file 2 for the original German wording). Figure 1 also includes all questions translated into English. The questionnaire was divided into three sections: individual prophylaxis, professional tooth cleaning, socio-demographic information.

#### Individual prophylaxis (IP)

GDPs were asked if any of their patients were undergoing MBA treatment. If so, they were asked for their opinion on the responsibility for IP during MBA treatment and if they generally adapt oral hygiene recommendations and IP sessions to special MBA needs. (If not, they continued with part 3.) The evaluation of oral hygiene recommendations included the following topics: tooth brushing duration, tooth brushing frequency, toothbrush type, tooth brushing technique, additional aids for mechanical and chemical plaque control.

#### Professional tooth cleaning (PTC)

The second part addressed their opinion on the responsibility for PTC during MBA treatment and the clinical management of PTCs in the general dental practice.

#### Socio-demographic information

Finally, information on age and gender of the participant was requested. In addition, respondents were asked if they are self-employed or employed by a practice and if they provide orthodontic treatment themselves.

### Data collection

In November 2022, all 2744 GDPs in the region of Hesse, Germany, were invited to participate in the questionnaire study. The invitation letter contained the questionnaire in paper form with an addressed envelope for the return (free postage) and a QR code with a link to an online version. The online questionnaire was created with the online survey tool LimeSurvey (LimeSurvey GmbH, Hamburg, Germany). A reminder was sent by e-mail at the end of February 2023. Both the invitation letter and the e-mail reminders were sent out by the “Kassenzahnärztzliche Vereinigung Hessen” (KZVH), the regional association of statutory health insurance-accredited dentists in Hesse. Data collection was stopped by the end of April 2023, approximately five months after the questionnaire had been sent out.

### Data management and statistics

First, the data from the paper questionnaires were manually transferred to Excel following the double-checking principle. This step was not necessary for the online version. The free-text answers were assigned to either existing or new categories by two independent examiners (W.P.-J., N.C.B.). The initial agreement rate was above 80% for all categories. In cases of disagreement, the examiners discussed the answers until mutual agreement was reached.

Statistical analysis involved descriptive statistics presented as absolute values and percentages. Age is given as mean and standard deviation. The statistical analysis was performed using SPSS version 29.0 (IBM, Ehningen, Germany). Euler diagrams were created with *eulerr* [25], an online app based on an R package.

## Results

A total of 1014 GDPs completed and returned the questionnaire, the response rate was 37.0%. The vast majority of respondents (89.3%, *n* = 906) used the paper questionnaire, while only 10.7% (*n* = 108) responded online. The mean age was 51.3 ± 9.9 years (paper: 51.6 ± 9.9 years; online: 49.0 ± 9.8 years). Further information about age, gender and professional status separated by paper and online questionnaire are provided in Table 1.

**Table 1:** Socio-demographic information

	total (*n* = 1014)	paper (*n* = 906)	online (*n* = 108)
	n (%)	n (%)	n (%)
**Gender**			
Female	494 (48.7)	440 (48.6)	54 (50.0)
Male	506 (49.9)	453 (50.0)	53 (49.1)
Other	5 (0.5)	5 (0.5)	0 (0.0)
Missing/invalid information	9 (0.9)	8 (0.9)	1 (0.9)
**Age** (years)			
26–35	67 (6.6)	56 (6.2)	11 (10.2)
36–45	224 (22.1)	196 (21.6)	28 (25.9)
46–55	311 (30.7)	271 (29.9)	40 (37.0)
56–65	328 (32.3)	303 (33.5)	25 (23.1)
66–75	51 (5.0)	49 (5.4)	2 (1.9)
Missing/invalid information	33 (3.3)	31 (3.4)	2 (1.9)
**Professional status**			
Self-employed	936 (92.3)	836 (92.3)	100 (92.6)
Employed	64 (6.3)	57 (6.3)	7 (6.5)
Missing/invalid information	14 (1.4)	13 (1.4)	1 (0.9)

Some questions should only be answered if certain conditions were met. This resulted in varying sample sizes for some answers. The questionnaire’s branching logic is illustrated in a flow diagram (Fig. 1).

### Individual prophylaxis (IP)

**Fig. 2:**
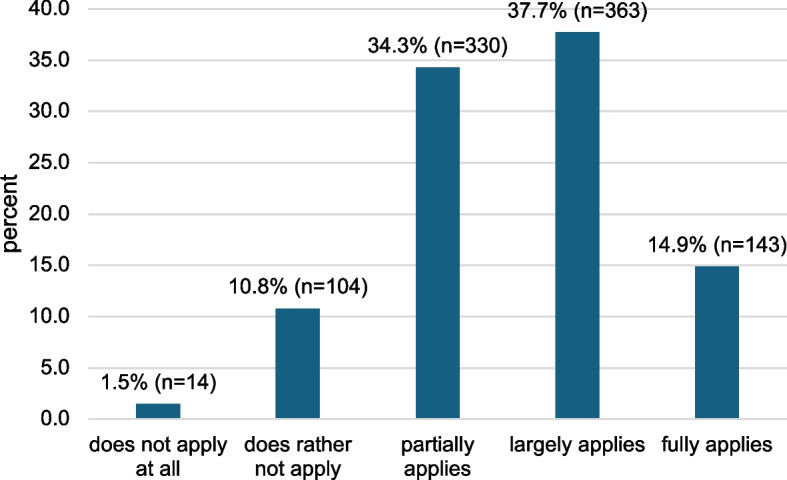
Evaluation of the following statement: *From my experience, MBA patients attend their IP sessions regularly (every 6 months) while undergoing orthodontic treatment.* Missing information (0.8%, *n* = 8) not shown

While only 7.9% (*n* = 76) of the respondents consider the orthodontist to be primarily responsible for IP in MBA patients, the majority considers the general dentist (45.8%, *n* = 441) or both (45.4%, *n* = 437) to be responsible for IP in MBA patients (missing information: 0.8%, *n* = 8). Around half of the respondents (52.6%) believe that MBA patients attend their IP sessions mostly or very regularly. 34.3% say that the sessions are only partially attended. In contrast, 12.3% have the opinion that patients tend not to attend the appointments or do not attend them at all (Fig. 2).

####  Oral hygiene recommendations

Most respondents (*n* = 922; 95,8%) answered that compared to patients without fixed appliances, MBA patients receive different oral hygiene recommendations during IP sessions. These recommendations were mostly related to a longer tooth brushing duration, followed by the use of additional tools for mechanical plaque removal (Table 2).

**Table 2:** Answers of GDPs who generally give MBA patients different oral hygiene recommendations than patients without an MBA during IP

	yes	no	missing
	n (%)	n (%)	n (%)
Do you recommend patients undergoing MBA treatment …			
to spend more time on oral hygiene than patients without MBA?	887 (96.2)	22 (2.4)	13 (1.4)
to brush their teeth more often than patients without MBA?	599 (65.0)	279 (30.3)	44 (4.7)
to use a different toothbrush than patients without MBA?	639 (69.3)	253 (27.4)	30 (3.3)
who use a manual toothbrush on daily basis a different brushing technique for brushing the buccal/labial surfaces than patients without MBA?	564 (61.2)	292 (31.7)	66 (7.1)
other cleaning aids besides the toothbrush for mechanical plaque removal than patients without MBA?	835 (90.6)	54 (5.9)	33 (3.5)
other antiplaque chemicals than patients without MBA?	418 (45.3)	458 (49.7)	46 (5.0)

#### Tooth brushing duration and frequency

**Fig. 3:**
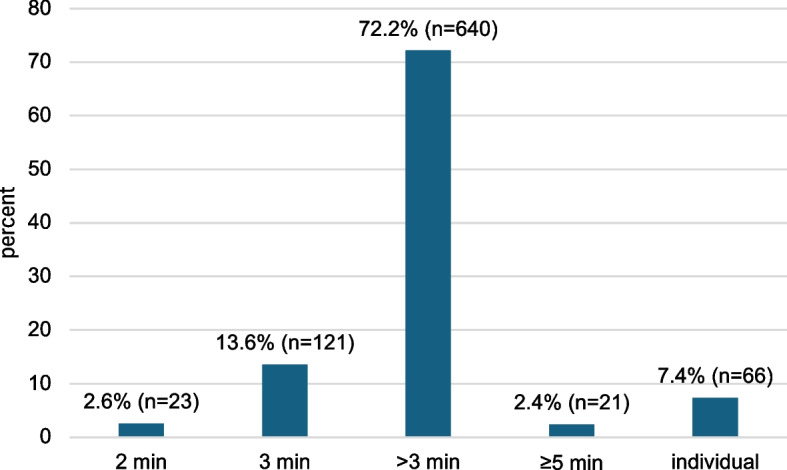
Recommended tooth brushing time for MBA patients. Missing/invalid information (1.8%, *n* = 16) is not shown

**Fig. 4:**
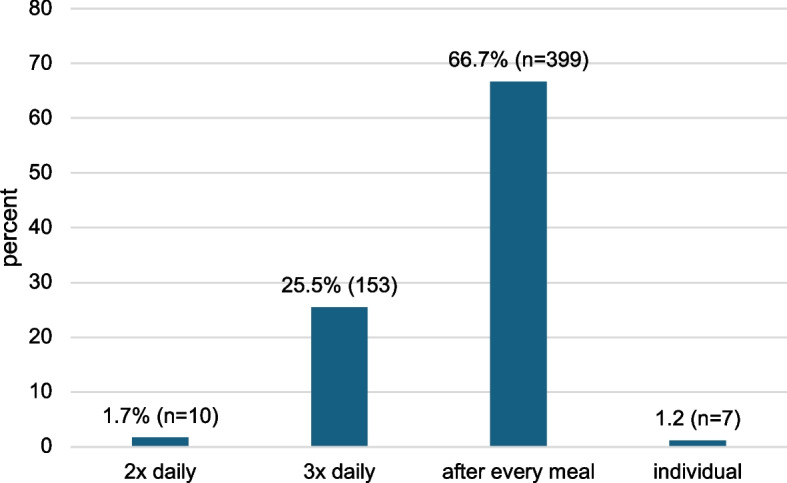
Recommended tooth brushing frequency for MBA patients. Missing/invalid information (3.3%, *n* = 20) is not shown

When asked how long MBA patients should spend brushing their teeth, 72.5% of GDPs suggest a brushing time of more than 3 min (Fig. 3). 7.4% do not provide a specific time recommendation: some of them indicate that the recommendation is individual or depends on the patient’s oral hygiene status, while others suggest that MBA patients should brush their teeth for as long as it takes to clean all areas of the mouth. 66.7% recommend tooth brushing after every meal, followed by 25.5% of GDPs, who suggest tooth brushing three times daily (Fig. 4).

#### Toothbrush

**Fig. 5:**
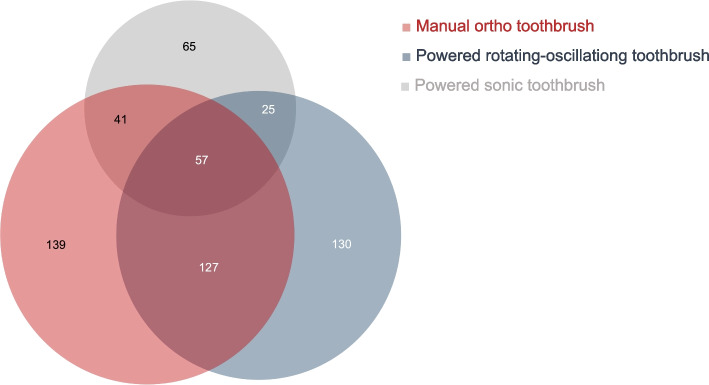
Recommended toothbrushes for MBA patients. The numbers indicate the number of GDPs. Missing/invalid information (*n* = 55) is not shown

Figure 5 shows the toothbrush recommendations for MBA patients. 21.8% (*n* = 139) of GDPs recommend a manual orthodontic toothbrush in general, 20.3% (*n* = 130) a powered rotating-oscillating, and 10.2% (*n* = 65) suggest a powered sonic toothbrush. 3.9% (*n* = 25) make a general recommendation for powered toothbrushes, regardless of their type of motion. Additionally, 19.9% (*n* = 127) recommend either a manual orthodontic toothbrush or a powered rotating-oscillating toothbrush. 8.9% (*n* = 57) do not recommend a specific brush, but rather base their recommendation on the patient's oral hygiene skills or individual preferences. 19 respondents also recommend a special orthodontic brush head for powered toothbrushes.

#### Tooth brushing technique

**Fig. 6:**
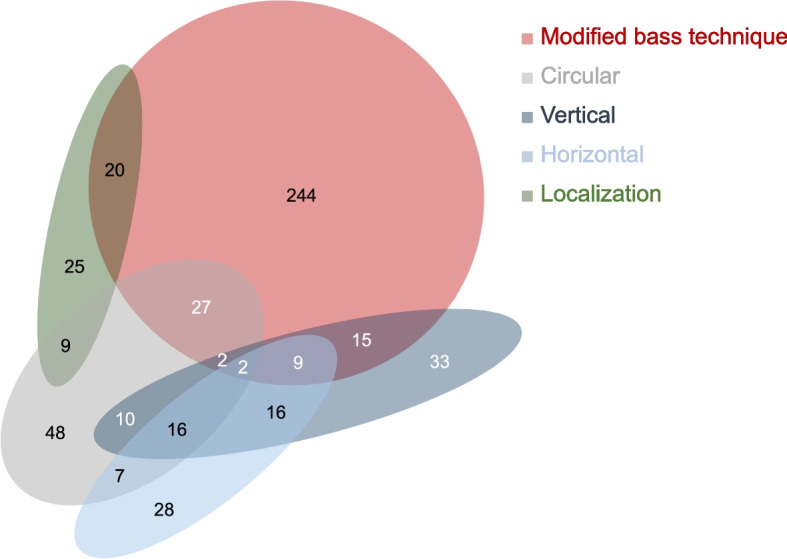
Recommended tooth brushing techniques for tooth outer surfaces for MBA patients. The numbers indicate the number of GDPs. Missing/invalid information (*n* = 25) is not shown. The following combinations of recommendations have been omitted for reasons of clarity: horizontal/localization *n* = 2, horizontal/modified bass technique *n* = 8, vertical/localization *n* = 3, circular/horizontal/modified bass technique *n* = 1, circular/horizontal/localization *n* = 1

The modified Bass technique is the most frequently recommended brushing technique to manual toothbrush users, either alone (43.3%, *n* = 244) or in combination with other techniques (14.9%, *n* = 84). Figure 6 shows the distribution of recommendations regarding the tooth brushing technique for the outer surfaces. The category “localization” does not describe a brushing technique per se, but several respondents recommend MBA patients to focus on explicit areas during tooth brushing: 8.7% (*n* = 49) recommend brushing the area above and below the brackets with the respective technique, and 2.0% (*n* = 11) suggest to focus on the gingival margin area. 12.3% of the respondents (*n* = 13) do not provide a general recommendation for a specific brushing technique but stated this to be individual.

#### Cleaning aids for mechanical plaque removal

**Fig. 7:**
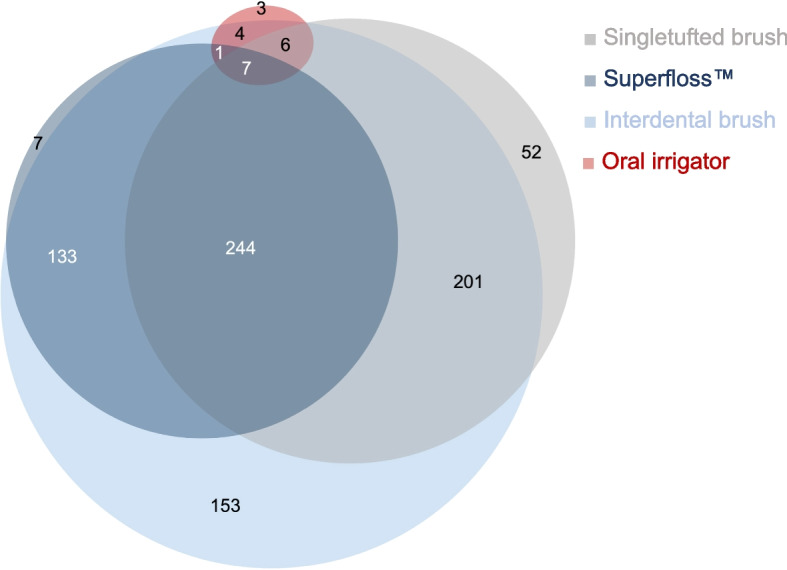
Recommended additional mechanical plaque control aids for MBA patients. The numbers indicate the number of GDPs. The combination of Superfloss™ and singletufted brushes (*n* = 23), interdental sticks alone (*n* = 1), respectively the combination of interdental sticks and other aids (*n* = 2) are not shown

0.8% (*n* = 7) of GDPs recommend Superfloss™, 6.2% (*n* = 52) a singletufted brush, 18.3% (*n* = 153) an interdental brush and 0.4% (*n* = 3) suggest the additional use of oral irrigators. 29.2% do not recommend a specific aid, but have listed a combination of Superfloss™, singletufted brush and interdental brush, some of them with the remark that the recommendation depends on the patient’s individual situation. The other combinations of aids for mechanical plaque control are shown in Fig. 7.

#### Antiplaque chemicals

**Fig. 8:**
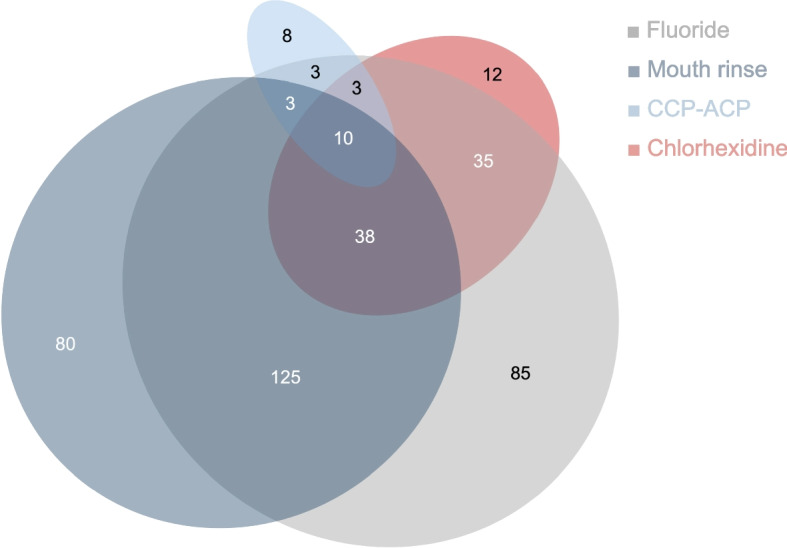
Recommended additional chemical plaque control aids for MBA patients. The numbers indicate the number of GDPs. The following recommended combinations are not shown: mouth rinse/CCP-ACP (0.4%, *n* = 2), mouth rinse/chlorhexidine (1.2%, *n* = 11), CCP-ACP/chlorhexidine (0.2%, *n* = 1), other (0.2%, *n* = 1). Invalid information (0.2%, *n* = 1) is not shown either

Figure 8 shows the recommendations for antiplaque chemicals. 20.4% (*n* = 85) of the respondents recommend the use of fluoride agents, 19.1% (*n* = 80) the use of mouth rinses; 1.9% (*n* = 8) recommend CPP-ACP products and 2.8% (*n* = 12) recommend chlorhexidine. Recommended combinations of these products are illustrated in Fig. 8. When recommending different combinations of products, 24 GDPs noted that these recommendations are individual. Occasionally, other recommendations, such as products containing apatite crystals or black cumin oil, were mentioned.

#### Visualization of dental plaque

When asked about alternative methods for visualizing dental plaque in MBA patients, 81.3% (*n* = 750) of respondents reported using none. In contrast, 14.9% (*n* = 137) used different techniques (missing information: 3.8%, *n* = 35). 43.8% (*n* = 60) of them use Mira-2-Ton®, while 13.1% (*n* = 18) use a fluorescent plaque disclosing agent. Additionally, 19.7% (*n* = 27) use a probe to show the plaque to the patient. 22.7% (*n* = 31) use various combinations of the methods listed above. Among these, two respondents mentioned that they combine these methods with an intraoral camera.

### Professional tooth cleaning (PTC)

Only 9.8% (*n* = 94) of the respondents consider the orthodontist as primarily responsible for PTC in MBA patients. The majority considers the general dentist (56.1%, *n* = 540) to be responsible and 32.8% (*n* = 316) of the respondents believe that the responsibility lies with both (missing information: 1.3%, *n* = 12). 73% (*n* = 702) recommend regular PTCs for MBA patients in addition to the IP sessions, and 65.8% (*n* = 633) perform them regularly in their practice. Of those who regularly perform PTCs, 17.2% (*n* = 109) remove the orthodontic archwires beforehand. Most of them (75.2%, *n* = 476) use powder water jet devices to remove plaque and calculus.

## Discussion

In Germany, public health insurance covers the cost of two prophylaxis sessions per year for children and adolescents. However, during orthodontic treatment, it is not clearly defined who is responsible for prophylaxis – the orthodontist or the GDP. A questionnaire study suggests that the orthodontist's awareness of oral hygiene practices during MBA treatment can be improved [26]. This raises the question of whether GDPs place greater emphasis on this issue. Therefore, the aim of the present questionnaire study was to evaluate GDPs' attitudes towards prophylaxis in patients undergoing MBA treatment and how they adapt prophylaxis sessions compared to patients without MBA.

In the present study, 2744 GDPs in Hesse received the questionnaire. Participants had the opportunity to respond online or in written form. The overall response rate was 37.0%, indicating a certain level of interest in this topic. In the literature, response rates for oral hygiene surveys among dental professionals range from 15.2% to 99.5% [27–29], depending on the study design. While Eichenauer et al. achieved a response rate of 99.5% using a telephone survey, the response rate for the web-based survey by Garyga et al. was only 15.2%. In our survey, the proportion of those who responded online was also low at 10.7%. Considering these findings, the postal method appears to be more promising despite increasing digitalization. This is also demonstrated by the questionnaire study by Baumer et al. who achieved a response rate of 52.4% using the postal method. The number of questions made a telephone survey appear impractical.

### Individual prophylaxis (IP)

The majority of respondents perceive the GDP as having primary (45.8%) or shared responsibility (45.4%) for IP during MBA treatment. However, the large proportion of perceived shared responsibility suggests room for improvement, as it requires good communication with the orthodontist. 46.6% of respondents believe that some or all of their patients do not attend IP sessions regularly or at all, which also indicates potential for improvement.

Most GDPs adapt oral hygiene recommendations to the special requirements of MBA patients. However, the evaluation of the oral hygiene recommendations shows a considerable variation.

#### Tooth brushing time

Most GDPs (72.5%) suggest a brushing time of more than 3 min, which exceeds the generally recommended minimum duration of 2 to 3 min for patients without MBA [30]. Koretsi et al. [31] assessed tooth brushing times in children and adults undergoing MBA treatment. Plaque was disclosed and participants were asked to brush their teeth with either a manual or powered toothbrush until staining was completely removed. Brushing times ranged from 2 min and 45 s to 3 min and 17 s with an average plaque reduction of 76%. The authors concluded that MBA patients can reach an effective plaque reduction in around three minutes, if the plaque is visible. These findings therefore correspond well with the recommendations regarding tooth brushing duration provided by most of our respondents. However, the visualization of plaque may present a limitation. Studies involving non-orthodontic patients, where plaque was not visible, indicate a significantly lower plaque reduction (approximately 35%) with brushing times exceeding 3 min [32, 33]. Plaque visualization in MBA patients can be particularly challenging, as many plaque disclosing agents tend to change the color of orthodontic elastics, alastics, and even the appliances themselves [34].

#### Tooth brushing frequency

The most common recommendation regarding toothbrushing frequency is to brush after every meal (66.7%), followed by brushing three times daily (25.5%). The evidence on the relationship between toothbrushing frequency and the development of new carious lesions is weak. Peros et al. [35] showed that brushing teeth after every meal (4 times a day) with a fluoride-containing toothpaste leads to a reduction in the concentration of *S. mutans* in saliva compared to brushing twice a day in MBA patients. Clinical parameters such as inflammation or plaque levels or the occurrence of WSLs were not recorded. Therefore, the clinical relevance of these results remains unclear.

#### Toothbrush

21.8% of GDPs recommend a manual orthodontic toothbrush to MBA patients in general, while 34.4% suggest a powered toothbrush. A powered toothbrush with rotating-oscillating technology is recommended more often than a sonic toothbrush. Whether powered or manual toothbrushes provide better result in terms of oral cleanliness is a controversial discussion to this date. For non-orthodontic patients the literature shows a small, but significant benefit of powered rotating-oscillating toothbrushes [36]. However, this effect is not shown for orthodontic patients. A systematic review by ElShehaby et al., [37] compared manual and powered toothbrushing during fixed orthodontic appliance treatment with regards to plaque and gingivitis. The use of a powered toothbrush did not demonstrate statistically significant short-term superiority. One possible explanation is that patients rely on the bristle movements of the powered toothbrush, although additional movements are necessary to effectively clean the areas around the brackets and under the archwire. However, this conclusion is drawn from only five studies. Approximately 35% of GDPs recommend either a manual orthodontic toothbrush or a powered toothbrush. The respondents rarely specified the criteria for their recommendations. Some GDPs noted that a powered toothbrush is suggested for patients with poor oral hygiene and others base their decision on the patient's preference, which appears more reasonable given the current evidence.

#### Tooth brushing technique

The modified Bass technique is the most frequently recommended brushing method for MBA patients using a manual toothbrush. In general, studies comparing the effectiveness of different tooth brushing techniques over a longer period face a significant limitation: it is not possible to assess how effectively participants adhere to the prescribed techniques, which is why the results must be interpreted with caution. A recent systematic review [38] compared the effects of different toothbrushing techniques on plaque and gingivitis. While there was limited evidence that adults taught the Bass technique show a reduction in plaque after brushing, this effect was not maintained in the long term. Notably, studies involving orthodontic patients were excluded from this review. Nassar et al. [39] compared the effectiveness of different tooth brushing techniques (scrubbing, modified Stillman, and Bass technique) in MBA patients. All groups demonstrated a reduction in plaque and gingivitis over the 9-month observation period. Only in terms of gingivitis reduction, the Bass group was superior to the others. However, this group showed the highest gingival index values at baseline, which was not statistically verified for comparability. At the end of the observation period, the gingival inflammation was at a similar level for all groups. Given this limitation, the superiority of the Bass technique over other methods remains questionable. Approximately 10% of GDPs suggest that MBA patients should focus on specific areas during toothbrushing, such as the gingival margin or the area beneath the archwire. An orthodontic brushing technique, which emphasizes the positioning of the bristles at the gingival margin, around the brackets, and beneath the archwire, demonstrated better plaque control compared to the modified Bass technique, particularly in the gingival and interproximal areas [40].

#### Mechanical aids

Interdental brushes were the most frequently recommended additional cleaning aids for mechanical plaque removal. An outdated Cochrane review highlights the lack of sufficient studies in this area. However, research suggests that interdental brushes can effectively reduce plaque [41]. Some authors recommend regularly adjusting the brush size during the course of MBA treatment to accommodate the changing interdental spaces [42]. Only one study demonstrates that interdental brushes are more effective at plaque removal than toothbrushing alone [43]. Following interdental brushes, single tufted brushes and Superfloss™ were the next most recommended options. To the authors' knowledge, studies on the effectiveness of single tufted brushes in MBA patients are currently lacking. One study [44] compared the effectiveness of Superfloss™ to the oral irrigator WaterPik®. Both cleaning aids were effective for plaque removal in patients undergoing orthodontic treatment when used in combination with toothbrushing. However, the lack of a control group prevents any conclusions about the superiority of these aids over toothbrushing alone. With approximately 2.5%, only a small portion of GDPs recommend the use of oral irrigators, which is consistent with existing evidence. Over a 4-week period, using a WaterPik® with an orthodontic tip improved plaque control in MBA patients compared to using a manual toothbrush alone [45]. However, this effect was not demonstrated over a longer period of 12 months [46].

#### Chemical products

In general, there is a consensus in the literature that fluoride-containing products reduce the risk of caries. It is therefore not surprising that the additional use of fluorides was the most frequently recommended additional chemical plaque control aid. A Cochrane review on fluorides for preventing WSLs during MBA treatment [7] found out that high fluoride foam applied professionally at each appointment throughout MBA treatment, might be effective in reducing the amount of orthodontic patients with new WSLs [8]. Another conclusion is that the patient use of a highly concentrated fluoride toothpaste throughout the MBA treatment might be more effective than the use of a conventional fluoride toothpaste [11]. The respondents recommended fluoride-containing mouth rinses more frequently than those containing chlorhexidine. Chlorhexidine has been found to reduce plaque accumulation and gingival inflammation during MBA treatment when compared to untreated controls. However, other mouthrinses containing probiotics or herbal ingredients have demonstrated similar effectiveness in managing gingivitis [47, 48]. Only a small proportion of GDPs (6.5%) recommend the use of casein phosphopeptide-amorphous calcium phosphate (CPP-ACP) products for MBA patients. Raphael and Blinkhorn [49] evaluated the effectiveness of Tooth Mousse® in the prevention and treatment of early dental caries in a systematic review. Three of the twelve RCTs included analyzed the caries-preventive effects in patients undergoing MBA treatment. The data show no significant advantage of using Tooth Mousse® products compared to brushing with fluoride toothpaste.

#### Visualization of dental plaque

A systematic review on the effectiveness of oral hygiene instructions with the aid of plaque-disclosing methods indicates a significant improvement on gingival inflammation in orthodontic patients but not on dental plaque [50]. Most GDPs neglected using alternative plaque-visualization methods. Most of those who use an alternative method reported using Mira-2-Ton®. Mira-2-Ton® has the negative side effect of discoloring elastics. Surprisingly, only a small number of GDPs use alternative options, such as fluorescent plaque disclosing agents, which counteract the latter issue.

### Professional tooth cleaning (PTC)

Most respondents perceive the GDP as having primary (56.1%) or shared responsibility (32.8%) for PTC during MBA treatment. The majority recommend additional PTCs and perform them regularly in the general dental practice. Regular patient motivation sessions and professional mechanical tooth cleaning by a dental hygienist, have proven to be effective in maintaining good oral hygiene during MBA treatment [23]. Although the removal of orthodontic archwires facilitates the mechanical tooth cleaning with polishing brushes or cups, only 17.2% of the GDPs report to do so prior to PTC. Given the risk of deforming the archwire if handled improperly, which could lead to undesired side effects, this approach seems reasonable. Powder water jet devices provide the advantage of efficient cleaning without damaging the MBA when used properly [51] and without the need to remove the archwire [24, 52].

### Limitations

One limitation of the present survey is that more than half of the GDPs in Hesse (63.0%) did not return the questionnaire. A nationwide survey of orthodontists in Germany on oral hygiene recommendations revealed significant regional differences regarding different oral hygiene products [27]. Since this survey was limited to one region, it is uncertain to what extent the results can be generalized to the rest of Germany. Another limitation might be the transferability of our results to other countries. Besides Germany, other European countries such as Denmark, the Netherlands, the United Kingdom, and Switzerland offer similar IP programs for children and adolescents free of charge [53]. In this context, our findings may not be exclusively relevant to Germany but could also provide insights applicable to other countries. One strength of the present study is the option to complete the questionnaire either online or in written form. Initially, a statistical comparison between online and written respondents was planned; however, it was omitted due to the low number of online responses and the similar age distribution among them. A limitation of content is that GDPs were not asked about nutrition recommendations, which are typically part of IP sessions. Although this is a key aspect of prevention, it covers a broad area. Including it in the questionnaire would have extended the completion time, potentially leading to a lower response rate. The questionnaire was explicitly directed at dentists. However, in everyday dental practice, individual prophylactic is often performed by dental assistants. While it can be assumed that they are trained by dentists, it remains uncertain whether the attitudes of dentists are conveyed to patients in exactly the same way.

## Conclusion

The majority of respondents perceive the general dentist as having primary or shared responsibility for IP and PTC during MBA treatment and adapt oral hygiene recommendations to the special requirements. However, the large proportion of perceived shared responsibility also suggests considerable room for improvement, as does the reduced attendance of IP sessions among MBA patients. Furthermore, the analysis showed a large range regarding oral hygiene recommendations. This indicates the need for standardized clinical guidelines.

## Supplementary Information

Supplementary Material 1.

Supplementary Material 2.

## Data Availability

The datasets used and/or analysed during the current study are available from the corresponding author on reasonable request.

## Abbreviations

IP: Individual prophylaxis
MBA: Multibracket appliance
WSL: White spot lesions
PTC: Professional tooth cleaning
GDP: General dental practitioner
KZVH: Kassenzahnärztliche Vereinigung Hessen
QR: Quick response
WPJ: Waldemar Petker-Jung (author)
NCB: Senior author
CPP-ACP: Casein phosphopeptide-amorphous calcium phosphate
RCT: Randomised controlled trial
SPSS: Statistical package for social sciences
