# Supplementary material for: Prophylaxis during multibracket appliance treatment – a survey among general dentists in Germany
Source: BMC Oral Health. 2025 Apr 2;25:472. doi: 10.1186/s12903-025-05843-4 (PMC11966794; doi:10.1186/s12903-025-05843-4)
Supplement: Supplementary file 1 — Supplementary Material 1. [file 12903_2025_5843_MOESM1_ESM.pdf]

Kassenzahnärztliche Vereinigung Hessen

JUSTUS-LIEBIG-UNIVERSITÄT GIESSEN

Questionnaire on prophylaxis in general dental practice for orthodontic patients

1. Individual prophylaxis (IP)

1. Are any of your patients undergoing MBA treatment (regardless of whether MBA was placed by yourself or by someone else)**?**

☐ Yes ☐ No → If not, please proceed to **section 3.**

2. How do you rate the following statement?

In my opinion, MBA patients attend IP sessions regularly (every six months) while undergoing orthodontic treatment.

☐ ------- ☐ ------- ☐ ------- ☐ ------- ☐

Fully applies / Largely applies / Partially applies / Does rather not apply / Does not apply at all

3. In your opinion, who is responsible for IP in MBA patients?

☐ Primarily the general dentist
☐ Primarily the orthodontist
☐ Both equally

4. Do MBA patients generally receive different oral hygiene recommendations than patients without MBA?

☐ Yes ☐ No → If not, please proceed to **section 2.**

The following questions aim to highlight the aspects in which oral hygiene recommendations differ for MBA patients.

5. Do you recommend patients undergoing MBA treatment to spend more time on oral hygiene than patients without MBA?

☐ No ☐ Yes

If yes, how much?

☐ 2 minutes ☐ 3 minutes ☐ > 3 minutes
☐ Other (please describe): ______________________

____

6. Do you recommend patients undergoing MBA treatment to brush their teeth more often than patients without MBA?

☐ No ☐ Yes

If yes, how often?

☐ 2x daily ☐ 3x daily ☐ After every meal
☐ Other (please describe): ______________________

____

7. Do you recommend patients undergoing MBA treatment a different toothbrush than patients without MBA?

☐ No ☐ Yes

If yes, which one?

☐ Powered rotating-oscillating toothbrush
☐ Powered sonic toothbrush
☐ Manual ortho toothbrush
☐ Other (please describe): ______________________

____

8. Do you recommend patients undergoing MBA treatment who use a manual toothbrush on a daily basis a different tooth brushing technique for brushing the buccal/labial surfaces than patients without MBA?

☐ No ☐ Yes

If yes, which one?

☐ Circular ☐ Vertical ☐ Horizontal
☐ Modified bass technique
☐ Other (please describe): ______________________

____

9. Do you recommend patients undergoing MBA treatment other cleaning aids besides the toothbrush for mechanical plaque removal than patients without MBA?

☐ No ☐ Yes

If yes, which ones?

☐ Superfloss ☐ Single tufted brush
☐ Interdental brush
☐ Other (please describe): ______________________

____

Please continue on the next page

10. Do you recommend patients undergoing MBA treatment other antiplaque chemicals than patients without MBA?

☐ No ☐ Yes

If yes, which ones?

☐ Mouth rinse ☐ Fluoride
☐ CCP-ACP ☐ Chlorhexidine
☐ Other (please describe): ______________________

____

11. Do you use other methods to visualize dental plaque in patients undergoing MBA treatment than in patients without MBA?

☐ No ☐ Yes

If yes, which ones?

☐ Mira-2-ton ☐ Probe
☐ Fluorescent plaque disclosing agent
☐ Other (please describe): ______________________

____

2. Professional tooth cleaning (PTC)

12. In your opinion, who is responsible for PTC of MBA patients?

☐ Primarily the general dentist
☐ Primarily the orthodontist
☐ Both equally

13. Do you recommend regular PTCs to your MBA patients in addition to IP sessions?

☐ No ☐ Yes

14. Do you regularly perform PTCs on MBA patients in your practice?

☐ Yes ☐ No → If not, please proceed to **section 3.**

15. Do you remove the orthodontic archwires before a PTC?

☐ No ☐ Yes

16. Do you use powder water jet devices?

☐ No ☐ Yes

3. General information

17. Gender

☐ Female ☐ Male ☐ Diverse

18. Age: ___________ years

19. I am …

☐ Self-employed ☐ Employed ☐ Junior dentist

20. Do you perform MBA treatment in your practice?

☐ No ☐ Yes

Thank you for your participation!

Please send us the completed questionnaire, **regardless of when you finished it**, using the enclosed envelope free of charge. Every return is important for data collection.
