# Supplementary material for: Prophylaxis during multibracket appliance treatment – a survey among general dentists in Germany
Source: BMC Oral Health. 2025 Apr 2;25:472. doi: 10.1186/s12903-025-05843-4 (PMC11966794; doi:10.1186/s12903-025-05843-4)
Supplement: Supplementary file 2 — Supplementary Material 2. [file 12903_2025_5843_MOESM2_ESM.pdf]

Fragebogen zur Prophylaxe in der allgemeinzahnärztlichen Praxis bei kieferorthopädischen Patienten

1. Individualprophylaxe (IP)

1. Umfasst Ihr Patientenstamm momentan MB-Patienten (unabhängig davon, ob Sie diese selbst eingesetzt haben oder ob diese extern eingesetzt wurde)**?**

☐ Ja ☐ Nein → Falls nein, bitte weiter zu **Abschnitt 3.**

2. Wie bewerten Sie die folgende Aussage?

Nach meinem Empfinden nehmen MB-Patienten während der KFO-Behandlung regelmäßig (6-monatlich) ihre IP-Untersuchungen war.

☐ ------- ☐ ------- ☐ ------- ☐ ------- ☐

Trifft voll zu | Trifft eher zu | Teils/teils | Trifft eher nicht zu | Trifft nicht zu

3. Wer ist Ihrer Meinung nach für die individualprophylaktische Betreuung von MB-Patienten zuständig?

☐ vor allem die allgemeinzahnärztliche Praxis
☐ vor allem die kieferorthopädische Praxis
☐ beide gleichermaßen

4. Erhalten in Ihrer Praxis MB-Patienten im Rahmen der IP im Allgemeinen andere Mundhygieneempfehlungen als Patienten ohne MB-Apparatur in situ?

☐ Ja ☐ Nein → Falls nein, bitte weiter zu **Abschnitt 2.**

Die nun folgenden Fragen beziehen sich auf die Mundhygieneempfehlungen für MB-Patienten.

5. Empfehlen Sie MB-Patienten mehr Zeit für die Mundhygiene einzuplanen als Patienten ohne MB?

☐ Nein ☐ Ja

Wenn ja, wie lange insgesamt?

☐ 2 Minuten ☐ 3 Minuten ☐ > 3 Minuten
☐ Sonstiges (bitte angeben): ____________________

____

6. Empfehlen Sie MB-Patienten sich häufiger die Zähne zu putzen als Patienten ohne MB?

☐ Nein ☐ Ja

Wenn ja, wie häufig?

☐ 2x täglich ☐ 3x täglich ☐ nach jeder Mahlzeit
☐ Sonstiges (bitte angeben): ____________________

____

7. Empfehlen Sie MB-Patienten andere Zahnbürsten als Patienten ohne MB?

☐ Nein ☐ Ja

Wenn ja, welche?

☐ elektrische rotierend-oszillierende Zahnbürste
☐ elektrische Schallzahnbürste
☐ manuelle Orthozahnbürste
☐ Sonstige (bitte angeben): ____________________

____

8. Empfehlen Sie MB-Patienten, die eine Handzahnbürste verwenden, eine andere Putztechnik zur Reinigung der Außenflächen als Patienten ohne MB?

☐ Nein ☐ Ja

Wenn ja, welche?

☐ kreisend ☐ vertikal ☐ horizontal
☐ modifizierte Basstechnik
☐ Sonstige (bitte angeben): ____________________

____

9. Empfehlen Sie MB-Patienten, neben der Zahnbürste, andere Hilfsmittel zur Plaqueentfernung als Patienten ohne MB?

☐ Nein ☐ Ja

Wenn ja, welche?

☐ Superfloss ☐ Einbüschelzahnbürsten
☐ Interdentalbürstchen
☐ Sonstige (bitte angeben): ____________________

____

Bitte weiter auf der nächsten Seite

10. Empfehlen Sie MB-Patienten andere chemische Hilfsmittel als Patienten ohne MB?
☐ Nein ☐ Ja
Wenn ja, welche?
☐ Mundspüllösung ☐ Fluoridlacke/-gele
☐ CCP-ACP-Präparate ☐ CHX-Präparate
☐ Sonstige (bitte angeben): ______________________

____

11. Nutzen Sie bei MB-Patienten andere Methoden zur Darstellung der Plaque als bei Patienten ohne MB?
☐ Nein ☐ Ja
Wenn ja, welche?
☐ Plaquetest Mira-2-ton ☐ Sonde
☐ Fluoreszierender Plaquetest
☐ Sonstiges (bitte angeben): ____________________

____

2. Professionelle Zahnreinigung (PZR)

12. Wer ist Ihrer Meinung nach für die PZR bei MB-Patienten zuständig?
☐ vor allem die allgemeinzahnärztliche Praxis
☐ vor allem die kieferorthopädische Praxis
☐ beide gleichermaßen

13. Empfehlen Sie Ihren MB-Patienten, neben den IP-Leistungen, regelmäßige PZRs?
☐ Nein ☐ Ja

14. Führen Sie in Ihrer Praxis regelmäßig PZRs bei MB-Patienten durch?
☐ Ja ☐ Nein → Falls nein, bitte weiter zu **Abschnitt 3.**

15. Werden die kieferorthopädischen Bögen vor einer PZR in der Regel entfernt?
☐ Nein ☐ Ja

16. Nutzen Sie für die Entfernung harter und weicher Beläge regelmäßig Pulverwasserstrahlgeräte?
☐ Nein ☐ Ja

3. Allgemeine Angaben

17. Geschlecht
☐ weiblich ☐ männlich ☐ divers

18. Alter: ___________ Jahre

19. Ich bin ...
☐ Selbstständige/r Zahnärztin/-arzt ☐ Angestellte/r Zahnärztin/-arzt ☐ Assistenzzahnärztin/-arzt

20. Führen Sie in Ihrer Praxis kieferorthopädische Behandlungen mit MB-Apparaturen durch?
☐ Nein ☐ Ja

Vielen Dank für die Teilnahme!

Bitte senden Sie uns den ausgefüllten Fragebogen, **unabhängig davon zu welchem Zeitpunkt Sie diesen beendet haben**, mit dem beigelegten Umschlag kostenlos zurück. Jede Rücksendung ist für die Datenerhebung von Wichtigkeit.
